# A78 “CHALLENGING PERSPECTIVES:” UNDERSTANDING CLINICIANS VIEWS ON MANAGING ALCOHOL USE DISORDER AND CIRRHOSIS

**DOI:** 10.1093/jcag/gwab049.077

**Published:** 2022-02-21

**Authors:** E Johnson, S M Ghosh, V J Daniels, T C Wild, P Tandon, A Hyde

**Affiliations:** 1 Medicine, University of Alberta, Edmonton, AB, Canada; 2 Medicine/Gastroenterology, University of Alberta, Edmonton, AB, Canada; 3 University of Alberta, Edmonton, AB, Canada

## Abstract

**Background:**

Alcohol use disorder (AUD) is one of the leading etiologies for liver cirrhosis and liver transplantation. Few individuals with AUD receive guideline-based care in the form of screening, brief intervention, referral to treatment, or prescription of anti-relapse pharmacotherapies. We interviewed clinicians across Alberta to assess the current experience and perceived barriers to managing AUD in people who have cirrhosis.

**Aims:**

The aim of this project is to summarize these findings to inform the development of an educational intervention.

**Methods:**

We used a qualitative descriptive approach to explore the experiences of clinicians who provide care for patients with cirrhosis and AUD in Alberta. We conducted semi-structured interviews directed by an interview guide. Interviews were recorded and transcribed verbatim. We used an inductive thematic analysis approach whereby transcripts were coded, with codes grouped into larger categories, then themes.

**Results:**

Sixteen clinicians participated in this study. Many participants acknowledged that they do not use a standardized approach to screening, brief intervention, and referral to treatment. Through thematic analysis we identified three themes surrounding barriers to managing AUD in patients with cirrhosis: (i) Practicing within knowledge constraints, (ii) Navigating limited resources and system challenges, and (iii) Acknowledging the complexity of patients who have cirrhosis and AUD.

**Conclusions:**

This research presents the perspectives of clinicians who manage people who have AUD and cirrhosis. Our results indicate that significant barriers exist that affect how clinicians manage AUD in the context of cirrhosis, including limited knowledge and resources, systemic challenges, and patient complexity. The information gathered in this investigation will be used to develop an accredited educational intervention that will delve deeper into these issues in order to have the greatest impact on clinicians who routinely interface with this patient population.

**Funding Agencies:**

Alberta Innovates Health Solutions

**Table:** 

Theme	Example Quote
Practicing within knowledge constraints	“In all the settings that I worked, [prescribing pharmacotherapy] is not something we do. And I realize that it is a knowledge deficit for us, and we need to get better.”
Navigating limited resources and systems challenges	“It’s a challenge in acute care because we only see them for a short snippet of time. We can’t build that relationship, follow it through, and see it to the end. We’re really trying to put a Band-Aid on something that needs an abdominal pad because we see them for such a short period.” “It’s not a drug I want to be prescribing myself from a practice management standpoint…because if there is an issue, they’re going to be able to see their PCP way easier than me.”
Acknowledging complexity of patients who have cirrhosis and alcohol use disorder	“A lot of my patients are very advanced cirrhosis…where my choices are very, very limited. I don’t see a lot of people now that are Child Pugh A …it’s just sort of the way the practice is.”

